# A collaborative endeavour to integrate leadership and person-centred ethics: a focus group study on experiences from developing and realising an educational programme to support the transition towards person-centred care

**DOI:** 10.1186/s12913-024-10793-8

**Published:** 2024-03-29

**Authors:** Qarin Lood, Eric Carlström, Charlotte Klinga, Emmelie Barenfeld

**Affiliations:** 1 Department of Health and Rehabilitation, Institute of Neuroscience and Physiology, Sahlgrenska Academy, University of Gothenburg, Box 455, SE-40530 Gothenburg, Sweden; 2 Centre for Person‑Centred Care (GPCC), University of Gothenburg, Gothenburg, Sweden; 3 Centre for Ageing and Health—AgeCap, University of Gothenburg, Gothenburg, Sweden; 4 School of Nursing and Midwifery, La Trobe University, Melbourne, Australia; 5 Institute of Health and Care Sciences, Sahlgrenska Academy, University of Gothenburg, Box 457, SE-40530 Gothenburg, Sweden; 6 School of Business, Campus Vestfold, University of South-Eastern Norway, Kongsberg, Norway; 7 Department of Learning, Informatics, Management and Ethics, Medical Management Centre, Karolinska Institutet, Stockholm, Sweden; 8 Research and Development Unit for Older Persons (FOU nu), Stockholm Health Care Services, Stockholm, Sweden

**Keywords:** Person-centred care, Person-centredness, Person-centred ethics, Leadership, Healthcare, Social care, Pedagogics, Curriculum development, Implementation

## Abstract

**Background:**

Ensuring the transition towards person-centred care is a growing focus in health and social care systems globally. Presented as an ethical framework for health and social care professionals, such a transition requires strong leadership and organisational changes. However, there is limited guidance available on how to assist health and social care leaders in promoting person-centred practices. In response to this, the Swedish Association of Health Professionals and the University of Gothenburg Centre for Person-Centred Care collaborated to develop an educational programme on person-centred leadership targeting health and social care leaders to support the transition towards person-centred care in Sweden. The aim with this study was to explore programme management members’ experiences from the development and realisation of the programme.

**Methods:**

Focus group discussions were conducted, involving 12 members of the programme management team. Data from the discussions were analysed using a structured approach with emphasis the collaborative generation of knowledge through participant interaction.

**Results:**

The analysis visualises the preparations and actions involved in programme development and realisation as a collaborative endeavour, aimed at integrating leadership and person-centred ethics in a joint learning process. Participants described the programme as an ongoing exploration, extending beyond its formal duration. Leadership was thoughtfully interwoven with person-centred ethics throughout the programme, encompassing both the pedagogical approach and programme curriculum, to provide leaders with tangible tools for their daily use.

**Conclusions:**

According to our analysis, we conclude that a person-centred approach to both development and realisation of educational initiatives to support person-centred leadership is essential for programme enhancement and daily implementation of person-centred leadership. Our main message is that educational initiatives on the application of person-centred ethics is an ongoing and collaborative process, characterised by an exchange of ideas and collective efforts.

**Supplementary Information:**

The online version contains supplementary material available at 10.1186/s12913-024-10793-8.

## Background

There is a growing emphasis on endeavours to establish health and social care systems, procedures, and practices that prioritise the importance of persons [1]. This indicates a need to delve into how to promote the principles of person-centred care (PCC). Conceptualised as an ethical framework that directs healthcare professionals in their daily responsibilities, PCC serves as a core care philosophy necessitating strong leadership and substantial structural and organisational adjustments [2]. As such, the implementation of PCC has been described as complex and challenging [3, 4], requiring collective efforts and partnerships between health and social care stakeholders [5]. Health and social care leaders have been described as key stakeholders in the implementation of PCC [6–8], but there is little guidance on how to educate leaders to take on the role of leading towards PCC.

According to Swedish law [9], the design and execution of health and social care interventions should be person-centred in terms of being determined in partnership with the person in need of care as far as possible. Nevertheless, there are challenges in determining how person-centred ethics can be seamlessly incorporated into routine care practices [10]. Even in countries known to practice PCC, like the United Kingdom and Sweden, there seem to be barriers for the implementation of PCC. For instance, Moore et al. [10] describe adverse consequences of organisational norms and role expectations, recommending the need for robust leadership and adaptive strategies to support the implementation process. It is therefore argued that a person-centred approach should permeate leaders’ and, managers’ actions and their way of being when leading towards PCC to achieve the change in organisational culture required for implementation of PCC [7, 11, 12]. Hereafter, this leadership approach is referred to as person-centred leadership, described previously as an intricate, relational, and dynamic context-based approach to leadership, aspiring to empower both co-workers and leaders [13]. Person-centred leadership is portrayed as translating the ethics of PCC into everyday leadership practice, promoting a person-centred atmosphere by establishing trust and responsibility, encouraging innovation, and potentiating cultural bearers among co-workers [12]. Moreover, person-centred leadership includes making use of relational competencies to facilitate a workplace culture based on partnerships in decision-making and collaboration between leaders, co-workers, and persons in need of care [8]. Establishing prerequisites to enable PCC is also raised as an essential element of person-centred leadership [12, 14]. Still, health and social care leaders have limited resources when it comes to leading in a person-centred way, and past research has recommended the development of educational curricula that emphasise person-centred leadership [6, 7, 10]. Previous educational programmes on PCC have mainly targeted health and social care professionals [4], and little is known on how educational curricula should be developed to promote a person-centred culture throughout health and social care organisations.

The University of Gothenburg Centre for Person-centred Care (GPCC) has developed three routines to facilitate the translation of person-centred ethics to healthcare practice: (1) Initiating a partnership—patient narrative, (2) working in partnership—shared decision-making, (3) safeguarding the partnership—documenting the narrative [15]. These routines have been evaluated in healthcare research, indicating effectiveness on individual as well as organisational levels [7]. What is not yet known is how these routines can be applied on leadership to facilitate the transition towards PCC in different health and social care contexts. In 2015, the Swedish Association of Health Professionals (SAHP, a trade union for registered nurses, midwives, radiographers, and biomedical scientists) and GPCC therefore decided to initiate the development of an educational programme on person-centred leadership, targeting health and social care leaders who are members of the SAHP. The programme has later been revised to adhere to societal changes and the needs and preferences of health and social care leaders. As one part of the scientific exploration of this programme (assessments of effects and significance will be reported in separate studies), the aim with the study was thus to explore programme management members’ experiences from the development and realisation of an educational programme on person-centred leadership. More specifically, we sought answers to the following research question: what preparations and actions were involved in developing and realising an educational programme to support leadership directed towards PCC?

## Methods

### Design

With the aim to explore programme management members’ experiences, this study had a social constructivist design, applying focus group methodology [16, 17]. This means that the study was built on a view that knowledge is co-created in interaction between participants who share their views and experiences in focus group discussions. More specifically, this meant that the participants in the study were encouraged to stimulate each other in discussions, to explore their shared experiences from developing and realising the educational programme. This approach to focus group research is suitable to uncover knowledge that is concealed but understood by participants [18] (e.g., tacit knowledge on pedagogical approach and leadership skills in programme development and realisation). Moreover, as described in the literature [16, 17], shared experiences are a powerful tool for expressing both positive and negative aspects of what is being studied, which is why focus groups were considered an appropriate method for the study, rather than individual interviews. The Swedish Ethical Review Authority approved the study (dnr. 2022-04052-01) and the Consolidated criteria for reporting qualitative research (COREQ) [19] were utilised when writing this report.

### Setting

The curriculum for the educational programme under exploration has been developed and revised in collaboration between researchers from GPCC and educators from SAHP, forming the programme management, to support the realisation of person-centred ethics in leadership across different health and social care organisations in Sweden. Admitting 40 health and social care leaders per year, the programme was initially provided between 2015 and 2019. After being put on hold between 2020 and 2021 due to the COVID-19 pandemic, the programme was revised to a more digital format in 2022, admitting 80 leaders per year. To be admitted to the programme, leaders had to be responsible for units or care activities targeting people in need of health or social care. Most leaders in the programme have been middle managers, but there have also been leaders with other leading positions within the Swedish health and social care system.

Incorporating blended learning, the programme illuminates person-centred ethics and leadership from various perspectives over a six-month period to support leaders in achieving the following learning outcomes:

Knowledge and understanding

- Summarise key foundational principles relevant to person-centred care and person-centred leadership.
- Explain what characterises a person-centred leadership and employee perspective within the own organisation, supported by course literature and proven experience.

Competence and skills

- Discover and define opportunities and areas of development regarding how person-centred ethics is expressed in current practice.
- Create a proposal for an action plan for a change process towards person-centred care or person-centred leadership.
- Apply person-centred principles during the implementation of a change process.

Judgement and approach

- Critically discuss how organisation, culture/structure within different contexts influence the conditions for person-centred care.
- From a leadership perspective, assess the implementation, results of the change process, as well as the need for further actions.
- Discuss central assumptions within person-centred ethics in relation to sustainable development.

**Fig. 1:**
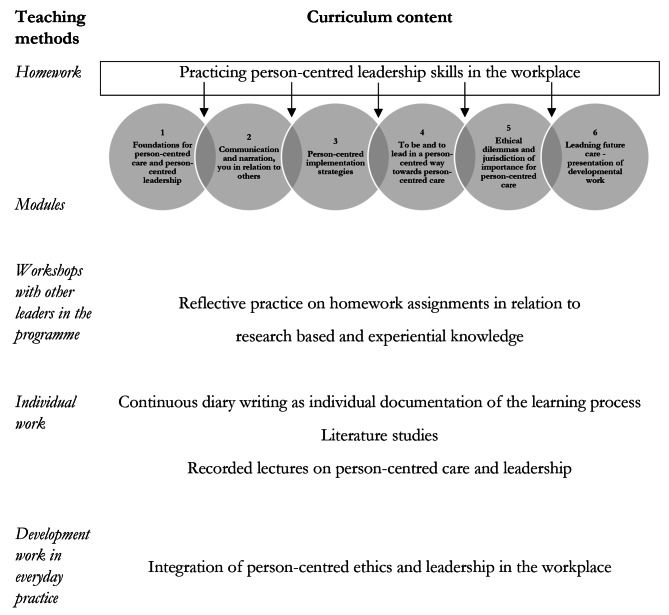
Overview of the educational curriculum

The programme corresponds to 7.5 higher education credits, divided into five digital modules (module 1, 3–6) and one physical module (module 2). Each module focuses on different aspects of person-centred leadership and person-centred ethics as follows: (1) Foundations for PCC and person-centred leadership, (2) communication and narration, you in relation to others, (3) person-centred implementation strategies, (4) to be and to lead in a person-centred way towards PCC, (5) ethical dilemmas and jurisdiction of importance for PCC, (6) leading future care—presentation of developmental work. Practical home-assignments to practice work in partnership were performed between the learning modules in both the original and revised programme. For an overview of the educational curriculum, please see Fig. 1.

### Participants

The participants were researchers with experience from studying PCC (*n* = 4) or leadership (*n* = 1) and educators from the SAHP (*n* = 7), all with experience of either developing and/or realising the programme. They were 11 females and one male, and they had been involved in different stages of the programme development and realisation between year 2015–2022. A total of four focus groups with three to four participants per group were conducted digitally with the participants taking part from their homes or offices during working hours. In line with the focus group methodology [16, 17], both homogeneity and heterogeneity were considered when selecting participants and putting together the groups. Homogeneity concerns having similar experiences and is important to generate discussion. In this study, homogeneity within each group was ensured by inviting persons with shared experiences of programme development phase and assignment. Heterogeneity concerns diversity within the target group and in this study, diversity was ensured by mixing participants with different work experience and roles in the development and realisation of the programme. Due to their roles in the development and realisation of the programme, two of the participants have been involved as co-authors (CK and EB), providing an insider perspective of the programme teaching methods and curriculum content that could not have been captured without their involvement. To ensure credibility of the findings they have not been involved in the primary analysis. Four persons in the programme management participated in two focus group discussions, with the aim to capture experiences from both the original development and realisation of the programme, and from the revision of the programme to a digital format. See Table 1 for details on participant roles. The names of participants referred to in this context are fictious to safeguard personal integrity and adhere to Swedish data protection regulations.

**Table 1:** Description of participants

Participant	Focus group(s)	Work organisation
Emma	1–2	SAHP*
Maria	1–2	SAHP*
Selma	1–2	University
Lisa	1–2	University
Fredrik	2	SAHP*
Anna	3	SAHP*
Eva-Britt	3	SAHP*
Tanja	3	SAHP*
Rakel	4	University
Sonja	4	University
Lena	4	SAHP*
Frida	4	University

* The Swedish Association of Health Professionals

### Procedure

Potential participants were invited via email, with a participant information statement and consent form attached. The statement comprised information on the aim of the study and what participation would require of participants should they choose to participate. All persons except one (who did not reply) consented to participate and were scheduled in for a digital focus group discussion using their preferred software (Microsoft Teams or Zoom). All focus groups were moderated by the second author and observed by a research amanuensis (group 1) or the first author (group 2–4) who took notes on the interaction between participants as well as each person’s engagement in the discussions.

Each focus group started with a confirmation of consent and a reminder to send the informed consent form to the researchers, followed by a short presentation of the participants and the researchers, including the participants’ current work role and their role in the development of the programme. Then, the moderator (second author) initiated the discussion by posing key questions developed by the research team involved in this study (see Supplementary file 1), starting with a question on why a leadership programme with focus on person-centred care was developed. Follow-up questions were posed to deepen the understanding of the participants’ experiences from the development and realisation of the programme. An important role for the moderator and the observer was to ensure that all participants were given an opportunity to speak, and to identify common elements in the discussions. The focus groups were audio recorded and transcribed verbatim by a professional transcription firm. Video was only used to stimulate interaction between participants and was not used in the analysis. Interaction was further facilitated by the moderator who encouraged participants to discuss their experiences with each other. The focus groups lasted between 57 and 100 min and were performed during 2022 and 2023.

### Data analysis

Krueger and Casey’s [20] systematic method for data analysis was used to analyse the audio recordings and transcriptions iteratively. This meant that the first author started the analysis procedure by listening to all focus group recordings and reading the transcripts and field notes carefully, making notes on content in relation to the study aim, to identify preliminary themes that were discussed with the other authors. Then, the first author started coding the transcribed data by sorting it according to the study aim and coding each response. To describe the content of the focus groups, the first author then prepared a summary statement that was discussed with all authors. The next step involved a formulation of themes. The summary statement was compared with the transcribed data and the field notes to identify internal consistency and the participants’ expressed experiences of importance of each question in terms of frequency, extensiveness, intensity, and specificity. This step resulted in revised themes and sub-themes that were discussed with all authors to reach a final interpretation of the meaning of the focus group discussions. The analysis was conducted in Swedish until the final formulation of themes and sub-themes was reached. The results were then translated to English.

## Results

**Fig. 2:**
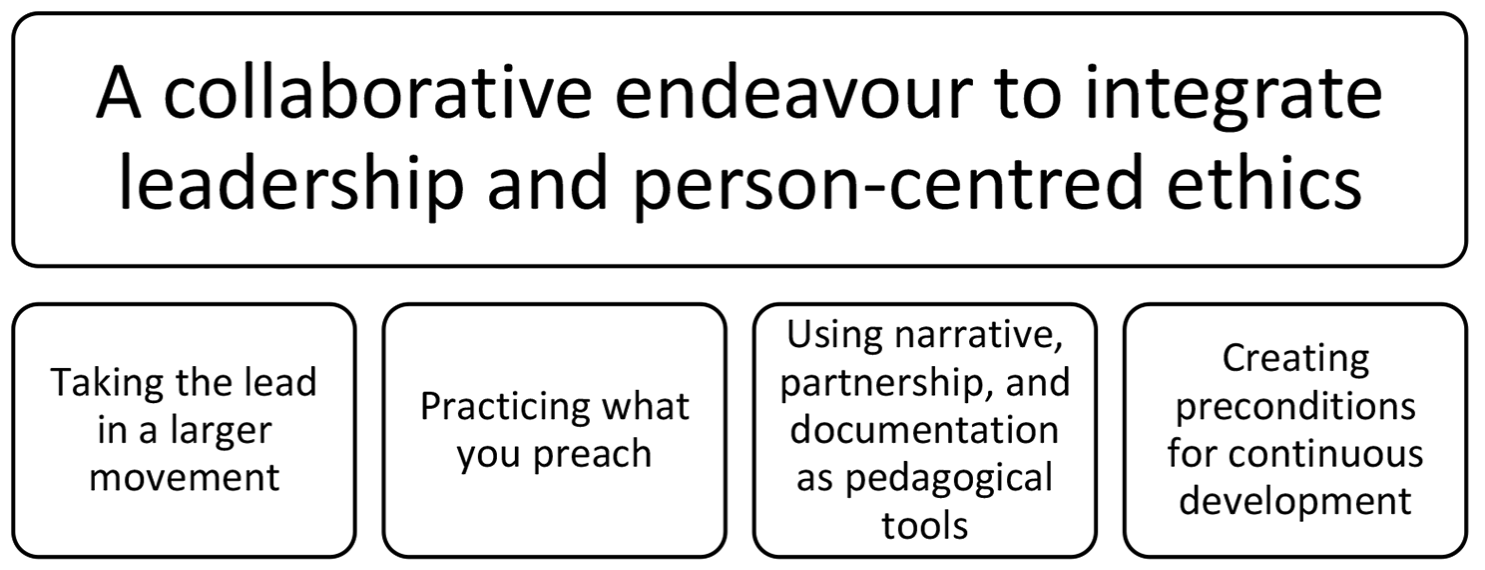
Overview of the thematical structure

The participants’ experiences from the development and realisation of the educational programme are described in the overarching theme “A collaborative endeavour to integrate leadership and person-centred ethics”, visualising a person-centred approach as essential in both preparations and actions involved in the development and realisation of the programme. These experiences are further described in four themes: (1) *Taking the lead in a larger movement, (2) Practicing what you preach, (3) Using narrative, partnership, and documentation as pedagogical tools*, and (4) *Creating preconditions for continuous development*, as visualised in Fig. 2 and described in detail below.

### A collaborative endeavour to integrate leadership and person-centred ethics

The overarching theme visualises the development and realisation of the educational programme as a dynamic and continuous process marked by collective efforts and an atmosphere of free exchange of ideas to allow for refinements of programme structure and content. Drawing from their experiences from academia, healthcare practice and leadership, the participants described how they developed the programme in partnership, to integrate knowledge on leadership and person-centred ethics. Used as both goals and means, person-centred ethics were thus experienced to have permeated the whole programme from development to realisation, with focus on joint learning among programme management as well as participating leaders. Dialogues between programme management and participating leaders were described as a pivotal element, allowing for joint discussions and reflections as a contrast to one-way communication through lectures. This was further described to foster partnerships between programme management and the participating leaders, which meant that the unique competencies and experiences of each person were harnessed to generate synergy effects during programme development, realisation and beyond.

### Taking the lead in a larger movement

With the goal to contribute to a deeper understanding of both what person-centred ethics entail, and how they can be integrated into leadership to change everyday care practice, the development of the programme was experienced to lead the way in the larger movement towards PCC in Sweden. The participants strove to be at the forefront of the larger movement, for example by admitting more leaders from different parts of the country in the revised, more digital version of the programme. Although there were some technical issues with the digital format, the benefits of reaching out geographically were experienced as facilitators for making it a joint course to lead Swedish health and social care organisations towards becoming more person-centred. Continuous reflection and openness to different persons’ perspectives during programme development and realisation were experienced as a support for both the participants’ own, as well as the leaders’, learning on how to facilitate the transition towards PCC. The participants also described how they came to realise that theoretical knowledge was not enough, practical knowledge on how to lead in a person-centred way was also needed. Practical exercises on who the leaders are and how they view themselves in relation to others were therefore created to deepen the participating leaders’ understanding of the significance of mutual respect and clarity around roles. These exercises were developed to assist leaders in pioneering person-centred practices for a broad spectrum of health and social care professionals, as envisioned in the quote from focus group one:

*Moderator: Well, the first question must be: Why a leadership education in person-centredness?*

*Maria:…the initial standpoint is that the Swedish Association of Health Professionals wants to take a leading role in the development of person-centred care… We want to contribute to the development of managers and leaders in healthcare, enabling them to work with person-centred approaches and also with more person-centred leadership… By investing in managers, a tremendous number of people will have the opportunity to benefit from this and will also be involved in the development and transformation towards more person-centred care.*

*Emma:…I think that we have seen that managers need support in this. There is a great interest in person-centred care, but how should it be implemented? How can one feel confident in person-centred care? What steps should be taken to make it a reality in the organisations? So, I believe it was a natural step to start with our managers and leaders.*

*Selma: What I find so exciting when looking at this question from sort of another perspective is that, from a research standpoint, we know that leadership is crucial for achieving implementation and sustainability in person-centred care. So, it feels, yes, so exciting and important that you have had and continue to have this education.*

### Practicing what you preach

During programme realisation, the participants described how they became aware of the need to practice what they preached in terms of having a person-centred approach towards leaders participating in the programme. Both knowing what PCC is, and being able to practice it through the programme’s pedagogical approach were described as important to facilitate each person’s learning process, illustrated by a quotation from focus group four:

*Lena: The actual pedagogy in the education was also about viewing them as persons. As you said Rakel,” What resources do you have?”. To also be very genuine in the person-ce(ntred) approach, it was not just knowledge that needed to be conveyed, it was also an… truly an attitude that needed to be embraced by those leading the programme.*

*Frida: Exactly. And I think that’s very… the pedagogical aspect of embodying— of also conveying…not necessarily living as one preaches, but also, what is a person-centred approach when you apply it to your own… where you stand? I believe… that was probably the challenging aspect to convey later in this bridge because it wasn’t explicitly stated that this would also be the task for those who became the carriers of culture from GPCC afterward.*

Practicing what you preach was further described in relation to the experienced need for transparency from programme management regarding programme organisation and pedagogical approach, i.e., putting words on what was done and how and why they were done, to clarify the thoughts and reasons behind it. Still, there were challenges described with the pedagogical approach, in striving to see each leader as a person and support their learning by listening to their narratives and acknowledging their individual resources and needs. As such, flexibility was a virtue emphasised as essential during programme realisation, allowing for different pedagogical methods, for plans to shift, goals to evolve, and perspectives to change.

### Using narrative, partnership, and documentation as pedagogical tools

This theme describes how the core concepts of GPCC’s model for PCC [15], i.e., *narrative, partnership*, and *documentation*, became key pedagogical tools to bridge experienced challenges with conveying how person-centred ethics could be applied in day-to-day leadership. The participants described how using *narratives* seemed to have sparked the leaders’ awareness of how they lead, which role they have within their organisation, and who they are in relation to other people. *Partnership* was experienced as a tool for leaders to understand how their relationships with co-workers had evolved by listening to, and acknowledging, them as persons. Practical exercises and examples were experienced to support the leaders in how narratives can be used to build partnerships with co-workers and come to shared decisions that are jointly documented. *Documentation* was also experienced as a pedagogical tool for joint reflection and learning among the participating leaders, through presenting and discussing their documented plans for how to integrate person-centred ethics with their leadership within their organisations. We have chosen a quotation from focus group two to visualise how GPCC’s model was used as a pedagogical tool during the programme.

*Selma: We have discussed partnerships based on the model you (the moderator) just described. We have provided practical examples throughout the entire education on how to work in partnership and how to use narratives and dialogue. How we arrive at shared decision-making, documentation. This is something we have integrated into the entire education that is present during each session. And to emphasise that it should be a mutuality in this partnership, where we see each other as persons with unique resources and abilities. But where we, at the same time, know that humans have a vulnerability, and that’s what allows us to open up to each other in a partnership.*

*Maria: That’s one part, I think. But it’s also… just as you said (moderator’s name), it is… being a manager also means having certain expectations placed on oneself that you should… So it’s… What should I say? This respect we have, the mutual respect we have for each other, understanding each other’s roles as well, I believe, is an important part of this person-centred leadership and partnership, making it clear to everyone what roles we have. And then it’s this with the competence and the person one is, with one’s entire life history in some way, that can be valuable to the organisation one is part of. And so I agree with what you are saying, Selma. But we also incorporate… Because Lisa has also talked about the fact that one needs… One needs a role where one needs to take on a different responsibility than what the employees may need as a manager.*

### Creating preconditions for continuous development

Supporting the collaborative endeavour towards PCC, the participants experienced a need for creating preconditions for continuous development of person-centred leadership after the programme has ended. One way of doing this was to cultivate a sense of shared purpose to leave an indelible mark on the leaders when it comes to person-centred ethics and becoming person-centred in their leadership. Even so, there was an experienced risk of leaders going back to working as before, without sustainable changes. To support leaders to go from knowledge to action, practical homework exercises were developed to provide the leaders with tools on a day-to-day basis, as described in previous themes. Fulfilling the expectation of participating leaders to take a leading role in the movement towards PCC, national networking also became part of the programme, to contribute to a sense of community and opportunities for continuous reflection and development. National networks of leaders and managers who have completed the programme were developed to provide an arena for sharing positive examples and mutual learning on how leadership and person-centred ethics can be integrated. Nevertheless, as many health and social care leaders have other professions than SAHP’s members, the participants expressed a need for continuous development of the programme to include all healthcare professions in the programme, to allow for a sharing of knowledge across professional boundaries. The importance of networks is described in the quotation from focus group three below.

*Tanja: The third (purpose of the programme) was to form networks within it. I mean, to create networks within this group and for ongoing work. So, you, you got, well, let’s put it this way, a national network of colleagues… whom I know continue to exchange thoughts and ideas with each other. I’ve met several of these participants now in… over the years and in my new assignments. And since there are 200 people, I can’t remember exactly which course. But they remember precisely. And they talk about how they have continued. And also that it has inspired them to take leadership positions in the transition towards integrated care.*

*Eva-Britt: Yes, I know that… think that last part is really important to emphasise as well, that you form networks and that it’s not just… just as you said, that it is the components, Tanja. You gain knowledge and abilities to find your motivations and so on. And it’s also part of that… that “I set the ball in motion both at home and the contacts I have across the country and so on.” It’s truly an active education that aims to bring about change in healthcare towards person-centredness.*

## Discussion

This study aimed to explore programme management members’ experiences from the development and realisation of an educational programme on person-centred leadership. Our main finding is the illustration of how person-centred ethics permeated the whole programme, from development to realisation. The participants highlighted the importance of dialogues and continuous reflection during programme development, to allow for innovative collaboration, mutual support, and a commitment to support leaders to go from knowledge to action. Overcoming challenges with communicating person-centred ethics, the participants further provided examples of how to apply GPCC’s routines for PCC (narratives, partnership and documentation) [15] as pedagogical tools to support a person-centred leadership. This knowledge can be used to develop educational curricula to support health and social care leaders in leading towards PCC.

To the best of our knowledge, there is a limited presence of educational curricula that genuinely embrace a person-centred pedagogical approach or are designed to educate health and social care leaders with a person-centred focus [21]. Previous research has highlighted that educational curricula should be characterised by innovation, not only in their preparation of practitioners but also in their proactive development of healthcare practice environments and cultures that promote PCC [22]. Björkman et al. [23] further describe the implementation of PCC in Swedish higher education of healthcare professionals as an ongoing and fragmented process, primarily led by persons with particular interests. Highlighting uncertainty and ambiguity concerning the significance and worth of PCC, as well as the methods for effective implementation, they suggest further research on the fundamental essence of PCC as an educational subject, alongside the development of suitable didactic strategies aimed at guiding students to become proficient in person-centred practice [23]. Our findings answer to this call for research and are especially relevant in the light of the paucity of research concerning the practical implementation of values within health and social care organisations, particularly in the realm of person-centred leadership [8]. A novel discovery from our study involves outlining factors to consider when creating person-centred curricula for leaders in health and social care. We recommend adopting a person-centred pedagogical approach, which utilises narratives, partnership, and documentation as tools for leaders to employ a person-centred approach in their leadership roles. Visualising person-centred ethics both as goals and means, the participants described the employment of a person-centred approach as an iterative learning process, facilitated by partnerships between programme management, participating leaders, and co-workers within the leaders’ organisations. This finding is supported by a recent international education initiative [24], illustrating the need for person-centred curricula to be both philosophically and methodologically aligned with person-centred principles. In agreement with this initiative, our findings suggest that person-centred curricula are needed to capture the intricacies of implementing PCC in contemporary healthcare organisations.

The interconnectedness between PCC and person-centred leadership has been described in previous literature [8], and implementation of PCC can be seen as a strategic healthcare system change. Such system changes can be very difficult [25] with contextual aspects shaping the change process in complex ways [26, 27]. For instance, change may be affected by the complex integration of local cultures, professional attitudes, communication patterns and leadership styles [28]. The studied programme was specifically developed to take the lead in the ongoing transition towards PCC in Sweden. Practical homework assignments were combined with theoretical lectures, literature studies and reflective practice to support the leaders’ learning process and provide them with everyday tools for person-centred leadership. The homework assignments were developed to allow leaders to experience person-centredness in their leadership roles. Put in relation to the existing literature on learning [29], this could be understood as second-degree learning, denoting a profound form of learning where collective values undergo transformation to the extent that they impact the person’s actual work. As described by Binns [30], leadership at the level of everyday practice is fundamentally relational and somewhat removed from management and hierarchical position [30]. Viewed from this perspective, second-degree learning is a crucial requirement for successful integration of person-centred ethics and leadership. Allowing leaders to experience person-centredness in actual encounters with co-workers, the homework exercises in the studied programme provided a sense of authenticity. However, planning suitable and effective homework exercises poses a significant challenge for educators, especially when the goal is to enhance preparedness for collectively addressing the complexity of implementing PCC. Consequently, the development of person-centred curricula for health and social care leaders has a key-role in bridging challenges to implementation and supporting the realisation of PCC in everyday practice.

The power of healthcare systems has been demonstrated in shaping the implementation of new working methods [25], such as PCC. For example, health and social care professionals might exhibit resistance to reforms that are seen as altering established work routines. This mirrors the influence of professionals and the strategies employed by them. As reflected in our findings, educational programmes on person-centred ethics could provide guidance for leaders to enact essential changes among co-workers within their organisations. McCormack et al. [31] further describe the need for ongoing support of a learning culture within healthcare systems to facilitate PCC [31]. The continuous development described in our findings could be seen as such a support, incorporating learning environments for healthcare leaders to cultivate collaborative practices. Person-centred leadership can be regarded a collaborative practice, rooted in caring for co-workers. The modules in the educational programme under exploration were developed to support leaders in their learning process, to recognise and acknowledge the resources of both them and co-workers. In combination with the fostering of national networks of peers, the educational programme was described as a safe environment for reflection on person-centred ethics in the hierarchical environments which healthcare leaders often find themselves in. This was believed to enable leaders to act in a person-centred way and maintain authenticity in their leadership roles, but the nature of this study does not allow for such conclusions. We therefore suggest and plan for further evaluations of the programme’s impact on participating leaders.

Finally, regarding the practical implications of our findings, we suggest continuous development and refinements of educational curricula to fully embrace person-centred ethics as both the goals and means. Notably, dialogues played a crucial role in promoting such development. Inclusive discussions and joint reflection are needed for both programme development and realisation, to support second-degree learning on how to integrate person-centred ethics and leadership on a day-to-day basis. The person-centred approach to both pedagogics and leadership in the educational programme fostered a strong partnership between programme management and leaders and was described as a foundation for partnerships between leaders and co-workers. In management research, there are indications that organisations are characterised by workplace partnerships that may impact practice. For instance, Ferris et al. [32] delineate leaders as persons with proficiency in effectively comprehending co-workers and leveraging this understanding to motivate them to align with organisational goals. This is corroborated by our findings, which illustrate the need for educational curricula to recognise the practicalities of how change is put to action, namely, through collaborative efforts in everyday practice.

### Methodological considerations

Social constructivist focus groups are particularly useful for generating rich, context-specific data. They allow participants to interact with one another, building upon each other’s responses and providing nuanced insights that might be missed in individual interviews or surveys [17]. This epistemological foundation for the study was thoroughly considered in relation to the involvement of two of the participants as co-authors of this manuscript (CK and EB). The risk of biased interpretations of the findings was carefully reflected upon in relation to the benefits of utilising their insider perspective from being part of the programme development and realisation for both describing the programme and for translating the findings to practical implications. Furthermore, as Krueger and Casey’s [20] method is built upon understanding collective understanding, rather than focusing on individual participants’ voices, the risk of biased interpretation was minimised.

To avoid the development of excessive uniformity within the groups, we took great care in composing the groups, aiming to strike a balance between heterogeneity and homogeneity [20]. What bound the participants in our study together (homogeneity) was their mutual involvement in the programme development. Considering group dynamics, we also attuned to power imbalances and divided participants according to their roles in programme development. Past research has demonstrated that when people with shared experiences come together, they can engage in discussions with a sense of companionship, knowing that others can relate to their experiences, thus promoting a spirit of sharing [16]. There is, however, always a risk in focus group studies, that one or a few participants dominate the discussion, while others remain silent. This may skew the results and prevent a full exploration of diverse viewpoints. During the focus groups we therefore attuned to dominant voices and encouraged everyone to interact to positively impact the authenticity of the responses.

Since our focus groups were conducted digitally to enable participants from all over Sweden to participate, we were careful to compose the focus groups with people who already knew each other to establish rapport and trust among participants. In contrast to limitations highlighted in the literature [33] we encountered no technical problems during conduct, and the digital context was not experienced as a disruption in the participants’ sharing of experiences. Nevertheless, digital platforms may limit the ability to observe non-verbal cues such as body language, which can provide valuable context and depth to responses in traditional face-to-face focus groups [34]. What we observed in our study was a limited fluidity of conversation, possibly due to the digital context. The moderator strove to create a comfortable and non-judgemental atmosphere to encourage interaction, but due to the digital platform, participants were not able to engage in spontaneous exchanges and took turns rather than sharing their experiences freely.

It is important to note that each focus group in our study had a limited number of participants.

However, methodological literature [17, 20] indicates that small groups of three to six participants are typically very dynamic, and that the quality of discussions are more influenced by participant engagement that the sheer number of participants. Despite issues with spontaneous exchanges described above, the participants in our study seemed to value the opportunity to participate in focus groups, leading to rich discussions where they openly shared their perspectives. It is also important to remember that findings from focus groups are context-specific, and even if we involved all eligible persons but one, the participants may not represent the diversity of perspectives within a larger population. With the aim to understand the participants’ shared experiences and provide insight into their articulation of knowledge [17], our findings thus provide insight into the perspectives of the specific participants involved.

## Conclusions

In the context of implementing PCC through leadership, our findings advocate for an integration of person-centred ethics and leadership through a person-centred approach throughout programme development and realisation. The person-centred approach nurtured strong partnerships between programme management and leaders, forming the basis for leaders to build partnerships with co-workers within their organisations. Our findings further support continuous development and refinement of educational curricula, with meaningful dialogues being described as essential for both programme enhancement and the daily realisation of person-centred leadership. By recognising and harnessing the distinct competencies and experiences of each person involved, the development and realisation of the programme were experienced to yield synergistic outcomes, both during and after its completion. In essence, the person-centred approach aimed to create a dynamic and supportive environment for both programme management and participating leaders, to successfully integrate person-centred ethics with leadership in an educational curriculum for person-centred leadership.

### Electronic supplementary material

Below is the link to the electronic supplementary material.

Supplementary Material 1

## Data Availability

The data generated and analysed during this study are not publicly available due to the information provided to the involved persons when obtaining their informed consent, stating that all attempts would be made to maintain their confidentiality. De-identified data are available are available from the corresponding author upon reasonable request to enable review and will be stored for 10 years from publication at the University of Gothenburg, Sweden. All data are covered by the Swedish Public Access to Information and Secrecy Act and a confidentiality assessment will be performed at each individual request.

## Abbreviations

PCC: Person-centred care
GPCC: University of Gothenburg Centre for Person-Centred Care
SAHP: The Swedish Association of Health Professionals
