# Supplementary material for: A collaborative endeavour to integrate leadership and person-centred ethics: a focus group study on experiences from developing and realising an educational programme to support the transition towards person-centred care
Source: BMC Health Serv Res. 2024 Mar 29;24:395. doi: 10.1186/s12913-024-10793-8 (PMC10979622; doi:10.1186/s12913-024-10793-8)
Supplement: Supplementary file 1 — Supplementary Material 1 [file 12913_2024_10793_MOESM1_ESM.docx]

### Supplementary file 1

**Key questions**

1. Why was a leadership programme with focus on person-centred care developed?
2. What are the aims with the programme, short-term and long-term?
3. How is the aim conveyed?
4. How has the programme been developed over time?
5. What importance does a person-centred leadership have in the programme?
6. How is partnership taught and practiced?
7. What are the benefits of the programme?
8. What are your visions for the programme in the future?
